# Improvement Effects of Different Afforestation Measures on the Surface Soil of Alpine Sandy Land

**DOI:** 10.3390/biology14020144

**Published:** 2025-01-30

**Authors:** Shaobo Du, Huichun Xie, Gaosen Zhang, Feng Qiao, Guigong Geng, Chongyi E

**Affiliations:** 1 College of Geographical Sciences, Qinghai Normal University, Xining 810008, China; wo827288809@163.com; 2 Qinghai Forest Ecosystem Observation and Research Station in the Southern Qilian Mountains, Huzhu 810500, China; 3 Key Lab of Medicinal Animal and Plant Resources of Qinghai-Tibetan Plateau in Qinghai Province, Qinghai Normal University, Xining 810008, China; qiaofnm@163.com; 4 College of Life Sciences, Qinghai Normal University, Xining 810008, China; 5 Key Laboratory of Extreme Environmental Microbial Resources and Engineering, Northwest Institute of Eco-Environment and Resources, Chinese Academy of Sciences, Lanzhou 730000, China; gaosenzhang@hotmail.com; 6 Qinghai Academy of Agriculture and Forestry Sciences, Qinghai University, Xining 810016, China; genggg-298@163.com

**Keywords:** desertification control strategy, sandy soil, physicochemical properties, enzyme activity, bacterial community

## Abstract

Desertification severely impacts soil environments, necessitating effective control measures to improve sandy soil. On the alpine sandy land of Gonghe Basin, taking bare land containing mobile sand dunes (LD) as a reference, surface soil undergoing four afforestation measures, namely *Salix cheilophil*a + *Populus simonii* (WLYY), *Salix psammophila* + *Salix cheilophila* (SLWL), *Artemisia ordosica* + *Caragana korshinskii* (SHNT), and *Caragana korshinskii* (NT80), was studied, with soil physicochemical properties and enzyme activity measured and the bacterial community structure analyzed using Illumina high-throughput sequencing. Compared to LD, all four afforestation measures significantly reduced the sand content, while increasing soil total carbon, total nitrogen, organic matter, alkali-hydrolyzable nitrogen, and available potassium. WLYY, SLWL, and SHNT significantly increased the surface soil total phosphorus and total potassium. Catalase, sucrase, urease, and alkaline phosphatase activities significantly increased under all four measures. Among them, the highest improvements were observed under SLWL, followed by WLYY. All treatments increased soil bacterial community richness, exhibiting significantly different bacterial community compositions to those in LD. Total phosphorus was the key physicochemical factor affecting the soil bacterial community structure, while enzyme activity was significantly correlated with the relative abundance of most major bacterial phyla. All measures improved the surface soil environment, with SLWL demonstrating the best improvement. The results provide valuable reference for sand prevention and control strategies in alpine sandy areas and offer a theoretical basis for the ecological restoration of sandy soil microenvironments.

## Simple Summary

This study was an investigation of the improvement effects of different afforestation measures on the surface soil of alpine sandy land. The results showed that all four examined afforestation measures significantly improved the particle composition, increased the soil nutrient content, and enhanced enzyme activity in the surface soil, with the greatest increase in soil nutrient content and enzyme activity observed under afforestation with *S. psammophila* and *S. cheilophila*, followed by *S. cheilophila* Schneid and *Populus simonii*, with surface aggregation also observed. The bacterial community composition of the surface soil under the four sand control measures differed from that of bare land containing mobile sand dunes, with increased bacterial community richness observed. Total phosphorus was the key physicochemical factor affecting the soil bacterial community structure, and enzyme activity was significantly correlated with the relative abundance of most major bacterial phyla. The results indicate that afforestation with *S. Psammophila* and *S. cheilophila* Schneid is recommended for improving and restoring the soil environment in alpine sandy areas, potentially leading to the recovery of damaged soil.

## 1. Introduction

Desertification, a land degradation process that occurs in regions with dry and sandy soils, is caused by the combined effects of arid, semi-arid, and semi-humid conditions, coupled with climate factors and human activity, and is one of the most severe environmental issues in the world today [1,2]. China is severely affected by land desertification, especially in its northern regions, where desertification is widespread and increasing rapidly, attracting widespread attention [3]. During the desertification process, the land gradually degrades, the water retention capacity drops, and soil nutrients become increasingly deficient. The process renders it difficult for plants to survive, further accelerating desertification and significantly affecting the ecological environment and social economies [4,5]. Therefore, it is crucial to implement and select appropriate sand control measures that can improve sandy soil and prevent further desertification. Soil physicochemical properties, enzyme activity, and bacterial community structure are three important indicators that can be used to evaluate the effectiveness of soil improvement in desertification control [6,7], and several studies have examined different measures for improving the soil. Su et al. [8] found that Caragana microphylla of different ages increased the moisture content, organic carbon, and total nitrogen content of the surface soil in the semi-arid Horqin sandy land of northern China, and the total carbon and nitrogen content in the soil significantly increased as the forest aged. Anhua et al. [9] studied Salix cheilophila afforestation at different ages and found that, compared to the control group, all three afforestation measures affected the nutrients and enzyme activities of soil in the Kubuqi Desert, proving significant soil improvement effects for the establishment of Salix cheilophila shelter belts in the Kubuqi Desert. Zhang et al. [10] conducted cluster analysis of bacterial communities and found significant differences between the microenvironments in a four-year gravel sand barrier + grass seed plot and a twelve-year gravel sand barrier + grass seed plot, indicating these factors could effectively improve the soil in the Yellow River source area. These studies highlight the key role of these indicators in evaluating the effectiveness of soil improvement measures for desertification control, and the simultaneous use of all three is expected to provide a more accurate and systematic evaluation of the effectiveness of sand control measures.

Alpine sandy land is a type of sand land found at high altitudes (2500 to 3700 m), in cold and dry climates with low vegetation coverage [11]. The Gonghe Basin is located in the transition zone between the Kunlun and Qilian Mountains on the Tibetan Plateau in China, at an altitude ranging from 2600 to 3400 m. The desertified land in this area is characterized by widely distributed low temperatures, a dry climate, and a short frost-free period, resulting in harsh natural conditions and relatively high desertification hazards, rendering it a typical alpine sandy area [12,13]. Under the influence of human activity and global climate change, the desertification in the Gonghe Basin, which is already a sensitive and fragile ecological environment, has worsened, seriously threatening the local economic and resource security [14]. Currently, various sand control measures, mainly artificial afforestation, have been implemented in the alpine sandy land of the Gonghe Basin, and many scholars have evaluated the effects of these measures on the soil [15,16]. However, most studies have evaluated soil improvement via soil physicochemical properties and neglected the important factors of enzyme activity and bacterial community structure, limiting the research findings. Therefore, in order to evaluate the improvement effect of different afforestation measures on the surface soil of alpine sandy land more comprehensively, this study systematically evaluated the effects of four afforestation measures on the surface soil of alpine sandy land in the Gonghe Basin, as follows: (1) Measuring the physicochemical properties and enzyme activities of soil in differently afforested areas and the bare land containing mobile sand dunes (LD) is of great significance for understanding how afforestation measures affect soil health, soil ecosystem stability, and soil quality improvement. These data can be used as a key indicator to evaluate the effectiveness of afforestation measures and provide a scientific basis for selecting suitable afforestation techniques and improving soil quality. (2) The analysis of soil bacterial community structure under different afforestation measures and LD, using high-throughput sequencing technology, is helpful to further understand how soil bacterial communities respond to changes in afforestation measures, to reveal the impact of afforestation measures on soil biodiversity and ecosystem function, and to provide an important basis for selecting appropriate ecological restoration strategies. (3) Mantel tests, RDA, and correlational heatmap analysis were used to explain the correlation between soil physical and chemical properties, enzyme activity, and bacterial community structure, so as to understand the most key physical and chemical factors affecting soil bacterial communities in the study area, which is of great significance for predicting ecosystem responses and promoting the sustainable development of soil ecosystems. The study aims to provide a reference for selecting desertification control measures with optimal ecological benefits in other alpine sandy areas and a theoretical foundation for enhancing the microenvironmental stability of alpine sandy lands.

## 2. Materials and Methods

### 2.1. Study Area Overview

The study area is located in the southern sand area of the Shazhu Yu Sand Control Experimental Forest in Shazhu Yu Township, Gonghe County, Hainan Prefecture, Qinghai Province (100°25′ E, 36°24′ N), which lies in the Gonghe Basin in the northeastern part of the Tibetan Plateau at an altitude of approximately 2880 m. The area is characterized by a high-altitude arid and semi-arid continental climate, with dry and cold winters and springs. The annual average temperature ranges from 2.0 to 3.3 °C, with average temperatures of −14.2 °C in the coldest month (January) and 18 °C in the hottest month (July) and significant temperature variation from day to night. The average annual precipitation is 264 mm, and the annual evaporation ranges from 1528 to 1937 mm. Rainfall is concentrated from July to September, accounting for 70% of the total annual precipitation. The frost-free period lasts around 90 days, and the area is frequently affected by westerly and northwesterly winds, resulting in severe wind erosion. The average annual wind speed is 2.7 m/s, and the soil types are mainly brown calcareous soil, chestnut calcareous soil, saline soil, and sandy soil, with sandy soil being predominant [17]. No natural forest grows in the study area; however, shrubs, mainly *Caragana korshinskii*, *Kalidium foliatum*, *Oxytropis aciphylla*, *Salsola arbuscula*, *Nitraria tangutorum*, and *Artemisia desertorum*, and herbaceous plants, such as *Leymus secalinus* and *Stipa capillata*, are present.

### 2.2. Soil Sample Collection

Field surveys and soil sample collection were conducted in the southern sand area of the Shazhu Yu Sand Control Experimental Forest in Gonghe County in late July 2024 (plant growing season). Using the surface soil of the bare land containing mobile sand dunes (LD) as a control, the surface soil from areas subjected to the following four afforestation measures were selected for study: *Populus euphratica + Salix cheilophila* (WLYY), *Salix psammophila + Salix cheilophila* Schneid (SLWL), *Artemisia desertorum + Caragana korshinskii* (SHNT), and *Caragana korshinskii* (NT80). All afforestation was performed via hole planting and all measures were established in 1980. Before afforestation, the habitats were all located on mobile sand dunes in the high-altitude sandy areas of the Gonghe Basin, with basically similar site conditions. The four afforestation measures have been relatively successful in the study area, with large-scale areas and representative effectiveness showing similar types of sand control (Table 1). Six random sample plots were set up in each afforested region, with 50 m × 50 m plots established in the SLWL and SHNT plots and 20 m × 20 m plots established in the relatively smaller WLYY and NT80 sample areas. The six sample plots in each area were located as far apart as possible, and five-point sampling was used to collect mixed soil samples from a depth of 0 to 10 cm and from a depth of 10 to 20 cm from each plot. A total of 60 soil samples were collected and each sample was divided into two portions, one of which was placed in a 10 mL sterile centrifuge tube and stored in liquid nitrogen for subsequent soil bacterial sequencing, and the other was used for soil physicochemical property and enzyme activity measurement.

### 2.3. Soil Physicochemical Properties and Enzyme Activity Measurement

A Mastersizer2000 laser particle size meter (Malvern Company, Malvern, UK), a PB-10 pH meter (Beijing Sartorius Scientific Instrument Co., Ltd., Beijing, China), a DDSJ-308F Conductivity Meter (Shanghai Yi Electrical Scientific Instrument Co., Ltd.—LEIMi, Shanghai, China), a Titrette titrator (Plander, Wertheim, German), an ultraviolet spectrophotometer UV-1900i (Shimadzu, Kyoto, Japan), a Fp6410-flame Photometer (Shanghai Yidian Analytical Instrument Co., Ltd., Shanghai, China), and a FlashSMART Element Analyzer (Thermo Scientific, Beijing, China) were utilized to determine soil physicochemical properties and enzyme activity.

Soil particle composition was determined using the laser diffraction method, with soil particles classified into clay (<0.002 mm), silt (0.02–0.05 mm), and sand (>0.05 mm) according to US standards [18]. Soil pH was measured using the potentiometric method at a ratio of 1:2.5 (soil–water, *w*/*v*); electrical conductivity (EC) was measured using the conductivity method at a ratio of 1:5 (soil–water, *w*/*v*); soil bulk density (SBD) and soil water content (SWC) were measured using the ring knife method; alkali-hydrolyzable nitrogen (AN) was determined using the alkaline hydrolysis diffusion method; available phosphorus (AP) was measured using the sodium bicarbonate extraction and molybdenum-antimony colorimetric methods; total potassium (TK) and available potassium (AK) were measured using the flame photometry method; total phosphorus (TP) was measured using the NaOH fusion and molybdenum-antimony colorimetric methods; (TC) total carbon and (TN) nitrogen were determined using the combustion method; soil organic matter (SOM) was measured using the potassium dichromate-concentrated sulfuric acid external heating method [19]; hydrogen peroxide enzyme activity was determined using the potassium permanganate titration method [20]; alkaline phosphatase activity was measured using the disodium phenyl phosphate colorimetric method [21]; urease activity was measured using the indole phenol acid colorimetric method [22]; and sucrose activity was determined using the 3,5-dinitrosalicylic acid colorimetric method [23].

### 2.4. 16S rDNA Extraction and Sequencing

Total bacterial DNA was extracted from the soil samples using a DNA purification kit (MagaBio Soil Genomic DNA Purification Kit, TransGen Biotech, Beijing, China), and DNA was detected using 1% agarose gel electrophoresis. The V3–V4 variable region of the soil bacterial 16S ribosomal RNA (rRNA) gene was polymerase chain reaction (PCR)—amplified using the universal primers 338F and 806R [24], with the reaction system comprising 5 × FastPfu Buffer (4 μL), 2.5 mM deoxyribonucleoside-5′-triphosphate (dNTPs) (2 μL), Forward Primer (5 μM), Reverse Primer (5 μM) (0.8 μL), FastPfu Polymerase (0.4 μL), bovine serum albumin (BSA) (0.2 μL), Template DNA (10 ng), and H_2_O, which was added to obtain a final volume of 20 μL. Pre-denaturation was performed at 95 °C (3 min), followed by denaturation at 95 °C (30 s), annealing at 52 °C (30 s), extension at 72 °C (45 s), and PCR run for 27 cycles, followed by a final extension at 72 °C (10 min). After amplification, PCR products were detected using 2% agarose gel electrophoresis, followed by fluorescence quantitative analysis, and mixed according to sequencing requirements. The library was constructed using the TruSeqTM DNA Sample Prep Kit (TransGen Biotech, Beijing, China), and sequencing was performed on the MiSeq PE300 platform.

### 2.5. Data Processing and Analysis

The MiSeq sequencing generated paired-end (PE) read data. Based on the overlap relationship between the PE reads, FLASH 1.2.11 software was used to splice the paired reads into a single sequence, and Fastp 0.19.6 software was used for both the quality control of the reads and splicing effects and to distinguish samples based on the barcode and primer sequences at the beginning and end of the sequence, allowing valid sequences to be obtained for each sample. Reads were classified, transformed, and compared with the corresponding species database to obtain taxonomic information and abundance information of bacteria in soil samples. Statistical package for the social sciences (SPSS) 27.0 software was used for analysis of variance (ANOVA) analysis and Duncan’s test was used to determine significant differences. The paired-sample *t* test was used to analyze the difference significance of two deep soil layers at the same point in the same index. Uparse 7.0.1090 software was used to perform operational taxonomic unit (OTU) clustering on the spliced sequences and was quality-controlled at 97% similarity, with sequences divided into multiple OTUs at the 97% similarity level. Alpha diversity indices were calculated using mothur 1.30.2 software, and a Kruskal–Wallis rank sum test and a one-way ANOVA were used to evaluate the differences in the relative abundance of major bacterial phyla in soils from different treatment areas. Principal coordinate analysis (PCoA) statistical analysis was performed using R language 3.3.1 software, and PCoA analysis plots, bar charts of soil bacterial community composition, and correlation heatmaps were constructed using the results. Based on the major bacterial phyla in different afforestation treatment areas and bare land surface soils, the pheatmap (1.0.8) package in R 3.3.1 was used to construct community heatmaps that could reflect the similarity in the soil bacterial communities at the phylum level in different treatment areas. The boot and stats packages were used to test and plot differences between index groups, and the vegan package was used to construct RDA analysis plots and Mantel test network heatmaps.

## 3. Results

### 3.1. Soil Particle Composition in the Surface Layer of Differentlt Afforested Areas

**Figure 1:**
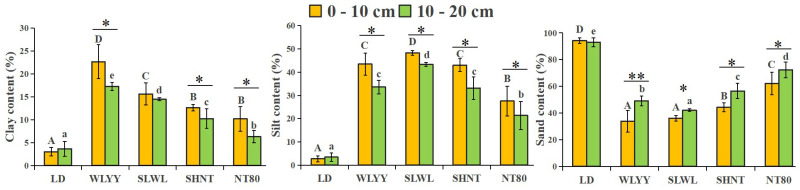
Soil particle composition. Different capital letters indicate significant differences for the same indicator in the 0–10 cm soil layers at different sites (*p* < 0.05). Different lowercase letters indicate significant differences in the 10–20 cm soil layers at different sites (*p* < 0.05). Asterisks indicate that, under the same index, there is a significant difference between the two deep soil layers at the same sample point: * 0.01 < *p* ≤ 0.05, ** 0.001 < *p* ≤ 0.01. LD: bare land containing mobile sand dunes; WLYY: *Salix cheilophila* + *Populus simonii*; SLWL: *Salix psammophila* + *Salix cheilophila*; SHNT: *Artemisia ordosica* + *Caragana korshinskii*; NT80: *Caragana korshinskii*.

The surface soil in the LD consists mainly of sand particles, which account for >92.90% of the total particle composition. However, the surface soil in the afforested areas is primarily composed of both silt and sand particles (Figure 1), with significantly higher clay and silt contents and significantly lower + sand content in the surface soil of the four afforested areas than in the LD. The clay content in the surface soil at different sampling points was WLYY > SLWL > SHNT > NT80 > LD, with silt content in the order SLWL > WLYY > SHNT > NT80 > LD, and significantly lower sand content in WLYY and SLWL than in the other areas. Compared to the 10–20 cm soil layers, the 0–10 cm soil layers demonstrated higher clay and silt contents and lower sand content.

### 3.2. Soil Physicochemical Properties in the Surface Layer of Differently Afforested Areas

**Figure 2:**
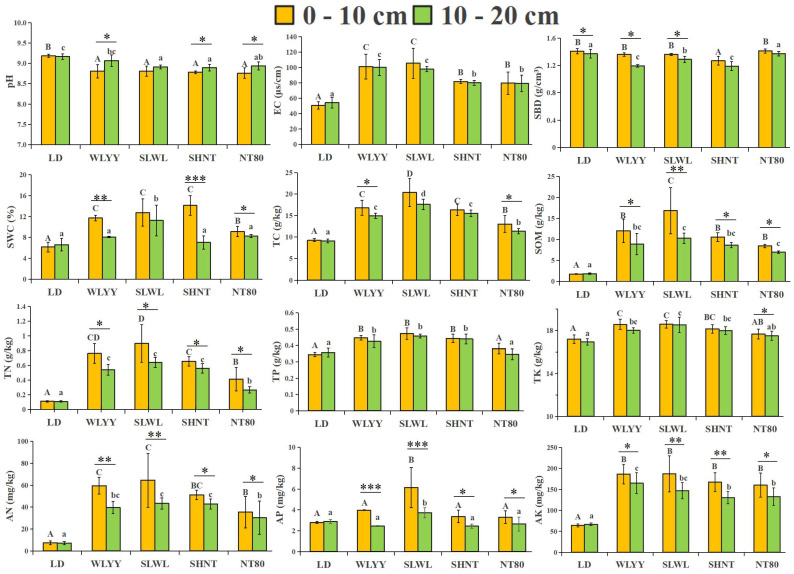
Soil physicochemical properties. Different capital letters indicate significant differences for the same indicator in the 0–10 cm soil layers at different sites (*p* < 0.05). Different lowercase letters indicate significant differences in the 10–20 cm soil layers at different sites (*p* < 0.05). Asterisks indicate that, under the same index, there is a significant difference between the two deep soil layers at the same sample point: * 0.01 < *p* ≤ 0.05, ** 0.001 < *p* ≤ 0.01, *** *p* ≤ 0.001. EC: electrical conductivity; SBD: soil bulk density; SWC: soil water content; TC: total carbon; TN: total nitrogen; SOM: soil organic matter; TP: total phosphorus; TK: total potassium; AN: alkali-hydrolyzable nitrogen; AP: available phosphorus; AK: available potassium; LD: bare land containing mobile sand dunes; WLYY: *Salix cheilophila* + *Populus simonii*; SLWL: *Salix psammophila* + *Salix cheilophila*; SHNT: *Artemisia ordosica* + *Caragana korshinskii*; NT80: *Caragana korshinskii*.

In terms of soil physicochemical properties, all four afforestation measures significantly improved the surface soil, but to varying degrees (Figure 2). The surface soil in the study area is alkaline, with pH values above 8.76. The pH values in the four afforested areas were all lower than those in the LD, while the EC was significantly higher in all afforested areas than in the LD. Significantly lower SBD was observed in the 0–10 cm soil layer of the SHNT treatment area compared to the LD, while no significant differences were observed in the SBD in the other treatments; however, in the 10–20 cm soil layer, the SBD was significantly lower in WLYY, SLWL, and SHNT than in the LD. The SWC was higher in all four treatment areas than in the LD; however, this was only significant for SLWL. The EC, SBD, and SWC were all higher in the 0–10 cm layer than in the 10–20 cm layer in all four treatment areas.

Compared to the LD, all four afforestation measures significantly increased the TC, TN, SOM, AN, and AK content of the surface soil, while WLYY, SLWL, and SHNT also significantly increased the TP and TK content in the surface soil, with the highest increases observed for SLWL, followed by WLYY. SLWL was also associated with significantly increased AP content in the surface soil. However, higher nutrient content was observed in the 0–10 cm layer compared to the 10–20 cm layer in all four treatment areas. These results indicate that all four afforestation measures improved the soil environment, but the effect varied by measure and soil depth.

### 3.3. Enzyme Activity in Surface Soils of Different Afforestation Measures

**Figure 3:**
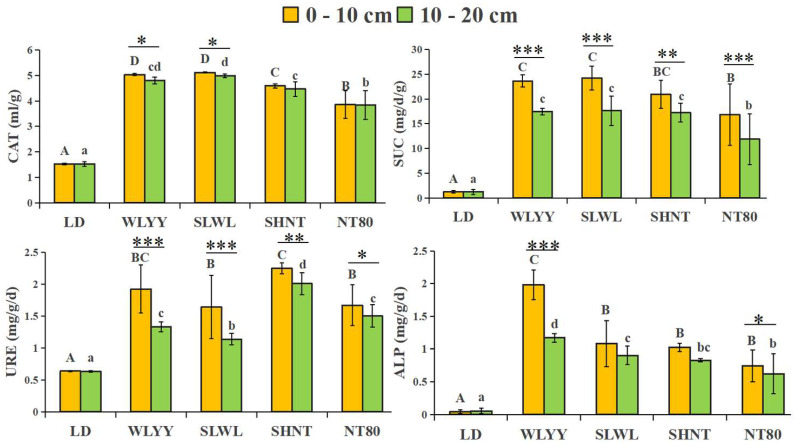
Soil enzyme activities. Different capital letters indicate significant differences for the same indicator in the 0–10 cm soil layers at different sites (*p* < 0.05). Different lowercase letters indicate significant differences in the 10–20 cm soil layers at different sites (*p* < 0.05); Asterisks indicate that under the same index, there is a significant difference between the two deep soil layers at the same sample point: * 0.01 < *p* ≤ 0.05, ** 0.001 < *p* ≤ 0.01, *** *p* ≤ 0.001. CAT: catalase; SUC: sucrase; URE: urease; ALP: alkaline phosphatase; LD: bare land containing mobile sand dunes; WLYY: *Salix cheilophila* + *Populus simonii*; SLWL: *Salix psammophila* + *Salix cheilophila*; SHNT: *Artemisia ordosica* + *Caragana korshinskii*; NT80: *Caragana korshinskii*.

Compared to the LD, significant improvements in the catalase, sucrase, urease, and alkaline phosphatase activity were observed in the surface soil under all four measures, with higher enzyme activity found in the 0–10 cm layer than that found in the 10–20 cm layer. This is similar to the comparison results of the soil physicochemical properties obtained for the two soil depth layers described in Section 2 (Figure 3). SLWL increased catalase and sucrase activity to a greater extent in the surface soils, while SHNT increased the urease activity more significantly and WLYY increased the alkaline phosphatase activity to a greater extent.

### 3.4. Correlation Between Soil Physicochemical Properties and Enzyme Activity

**Figure 4:**
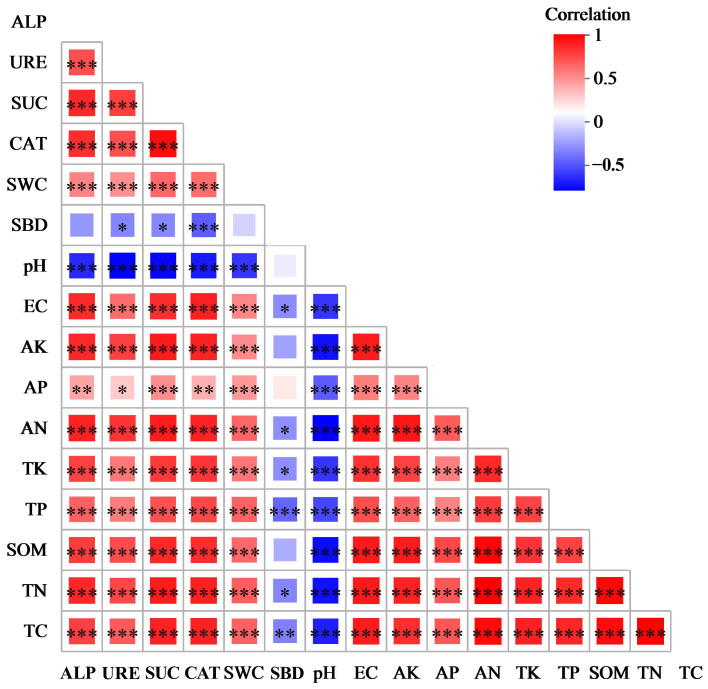
Heatmap showing correlation between soil physicochemical properties and enzyme activity. Different colors represent positive and negative correlations, with depth of color indicating correlation strength. Asterisks indicate significance: * 0.01 < *p* ≤ 0.05, ** 0.001 < *p* ≤ 0.01, *** *p* ≤ 0.001. EC: electrical conductivity; SBD: soil bulk density; SWC: soil water content; TC: total carbon; TN: total nitrogen; SOM: soil organic matter; TP: total phosphorus; TK: total potassium; AN: alkali-hydrolyzable nitrogen; AP: available phosphorus; AK: available potassium; CAT: catalase; SUC: sucrase; URE: urease; ALP: alkaline phosphatase.

The Mantel test was used to analyze the correlation between the soil physicochemical properties and enzyme activity in the surface soil in the differently afforested areas and on bare land (Figure 4). The pH value was significantly negatively correlated with other soil physicochemical properties and enzyme activity, except for SBD. Four of the studied enzymes were significantly positively correlated with the soil physicochemical properties, except for pH and SBD, and significant positive correlations were observed between the four studied enzymes in terms of activity, underscoring the importance of soil enzyme activity as an evaluation of soil quality. SWC was significantly positively correlated with EC and soil nutrient content and negatively correlated with SBD; however, these differences were not significant. SBD showed significant negative correlations with EC, AN, TP, TK, TN, and TC, while EC was significantly positively correlated with the soil nutrient content.

### 3.5. Soil Bacterial Community Structure in Different Afforestation Measures

#### 3.5.1. OTUs Statistical Analysis

**Figure 5:**
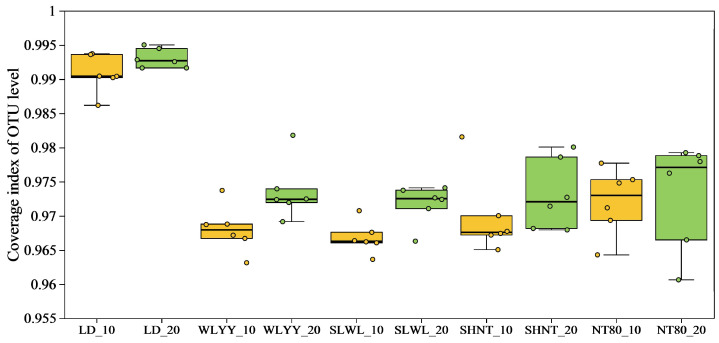
Soil bacterial community coverage. LD: bare land containing mobile sand dunes; WLYY: *Salix cheilophila* + *Populus simonii*; SLWL: *Salix psammophila* + *Salix cheilophila*; SHNT: *Artemisia ordosica* + *Caragana korshinskii*; NT80: *Caragana korshinskii*; _10: 0–10 cm soil layer of the sample; _20: 10–20 cm soil layer of the sample.

A total of 5,339,673 quality-controlled sequences were obtained from the samples, with an average of 88,995 sequences per sample. After clustering and classification, 27,180 OTUs were identified, belonging to 1 Kingdom, 47 Phyla, 158 Classes, 409 Orders, 688 Families, and 1356 Genera. The coverage of all samples was above 0.96 (Figure 5), indicating that the sequencing results accurately represent the true bacterial community structure in the surface soil of the study area.

#### 3.5.2. Soil Bacterial Alpha Diversity

**Figure 6:**
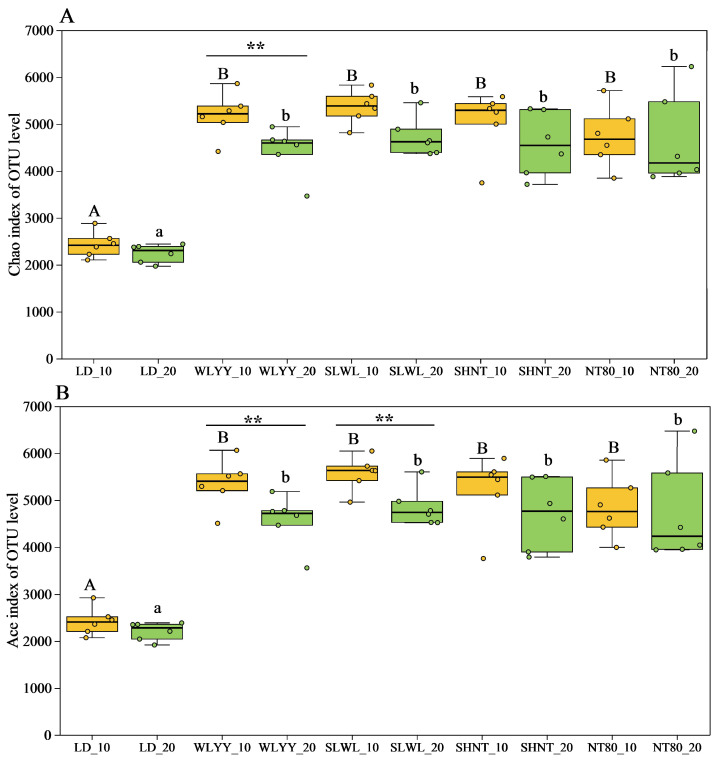
Soil bacterial community richness: (**A**) Chao and (**B**) Ace indices of OTU level. Different capital letters indicate significant differences for the same indicator in the 0–10 cm soil layers at different sites (*p* < 0.05). Different lowercase letters indicate significant differences in the 10–20 cm soil layers at different sites (*p* < 0.05). Asterisks indicate that, under the same index, there is a significant difference between the two deep soil layers at the same sample point: ** 0.001 < *p* ≤ 0.01. LD: bare land containing mobile sand dunes; WLYY: *Salix cheilophila* + *Populus simonii*; SLWL: *Salix psammophila* + *Salix cheilophila*; SHNT: *Artemisia ordosica* + *Caragana korshinskii*; NT80: *Caragana korshinskii*; _10: 0–10 cm soil layer of the sample; _20: 10–20 cm soil layer of the sample.

The Chao and Ace indexes are important indicators that reflect the richness of a soil bacterial community and were thus used to describe the soil bacterial communities in the surface soil of the differently afforested areas and bare land in this study (Figure 6). The results showed significantly higher Chao and Ace indices for the soil bacterial communities in the four afforested areas compared to the LD soil, indicating that afforestation can significantly increase the bacterial community richness at the surface of alpine sandy soils. The higher Chao and Ace indices in the 0–10 cm layer compared to the 10–20 cm layer indicate that the afforestation measures had greater impact on increasing the bacterial community richness in the upper soil layers.

**Figure 7:**
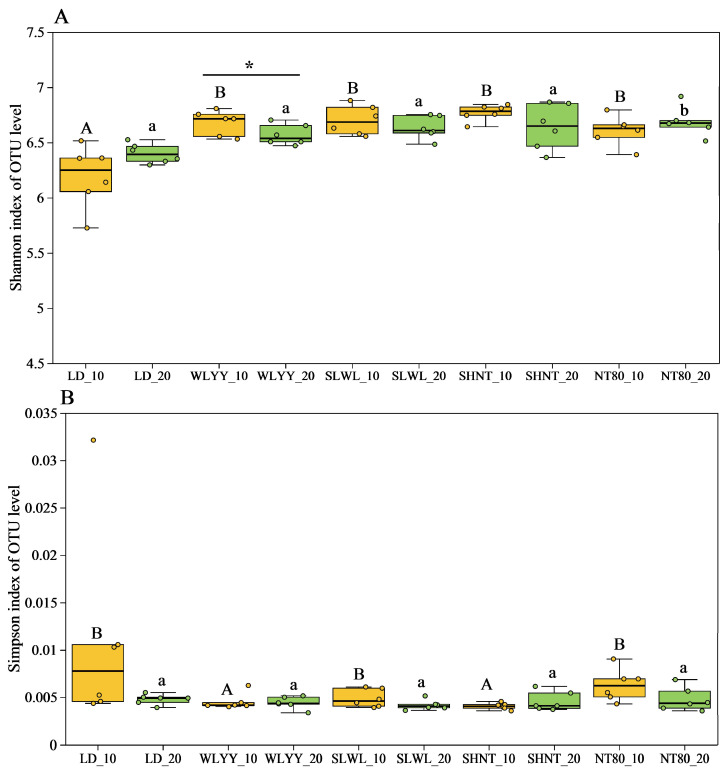
Soil bacterial community diversity: (**A**) Shannon and (**B**) Simpson indices of OTU level. Different capital letters indicate significant differences for the same indicator in the 0–10 cm soil layers at different sites (*p* < 0.05). Different lowercase letters indicate significant differences in the 10–20 cm soil layers at different sites (*p* < 0.05). Asterisks indicate that, under the same index, there is a significant difference between the two deep soil layers at the same sample point: * 0.01 < *p* ≤ 0.05. LD: bare land containing mobile sand dunes; WLYY: *Salix cheilophila* + *Populus simonii*; SLWL: *Salix psammophila* + *Salix cheilophila*; SHNT: *Artemisia ordosica* + *Caragana korshinskii*; NT80: *Caragana korshinskii*; _10: 0–10 cm soil layer of the sample; _20: 10–20 cm soil layer of the sample.

The Shannon and Simpson indexes are important indicators of soil bacterial community diversity and were thus also obtained for the soil bacterial communities in the surface soils of the differently afforested areas and compared to those from the LD (Figure 7). The results showed a significantly higher Shannon index for the soil bacterial communities in the 0–10 cm layer in areas subjected to the four afforestation measures compared to the LD, while the Simpson index for soil bacterial communities was significantly lower under the WLYY and SHNT measures compared to the LD. The Simpson index for the other areas was also lower than that observed in the LD; however, the differences were not significant. The fact that no significant differences were observed in either the Shannon or the Simpson indexes for soil bacterial communities in the 10–20 cm layer compared to the LD indicates that the afforestation measures primarily increased the bacterial community diversity in the 0–10 cm layer, especially WLYY and SHNT, where the increase in bacterial diversity is more significant.

#### 3.5.3. Composition of Soil Bacterial Communities

**Figure 8:**
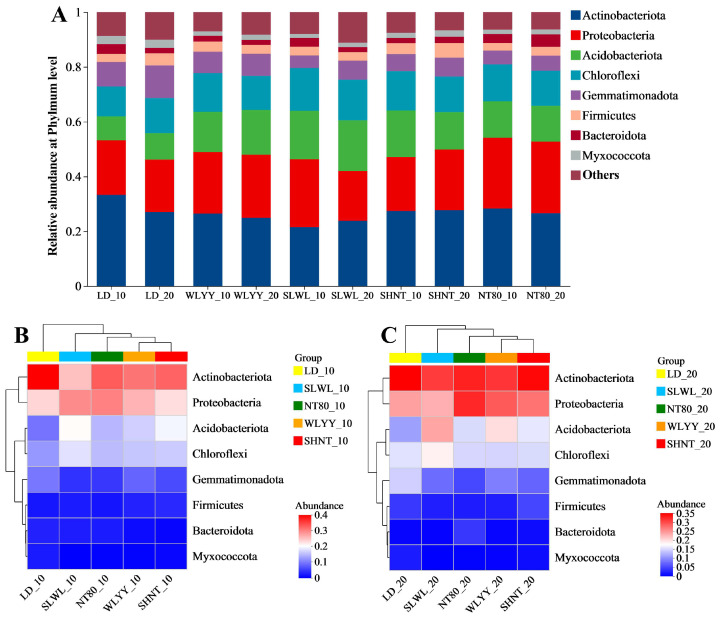
(**A**) Soil bacterial community composition and community (**B**,**C**) heatmaps. In (**A**), “Others” refers to bacterial phyla with a relative abundance greater than 8, sorted from high to low. In (**B**,**C**), different color blocks represent relative abundances of different species in samples. LD: bare land containing mobile sand dunes; WLYY: *Salix cheilophila* + *Populus simonii*; SLWL: *Salix psammophila* + *Salix cheilophila*; SHNT: *Artemisia ordosica* + *Caragana korshinskii*; NT80: *Caragana korshinskii*; _10: 0–10 cm soil layer of the sample; _20: 10–20 cm soil layer of the sample.

The soil bacterial community in the surface soil of the study area was mainly composed of eight bacterial phyla (Figure 8A), with Actinobacteriota, Proteobacteria, Acidobacteriota, and Chloroflexi dominating, accounting for 21.49 to 33.31%, 18.14 to 26.13%, 8.70 to 18.55%, and 10.91 to 15.63% of the total, respectively. Compared with the LD, both WLYY and SLWL led to reductions in the relative abundance of Actinobacteriota, Gemmatimonadota, Bacteroidota, and Myxococcota in the surface soil, while WLYY was associated with an increased relative abundance of Proteobacteria and Acidobacteriota, and SLWL with increased Acidobacteriota and Chloroflexi in the surface soil. SHNT reduced the relative abundance of Gemmatimonadota and Myxococcota, while increasing the relative abundance of Acidobacteriota, Chloroflexi, and Firmicutes, while NT80 reduced the relative abundance of Actinobacteriota, Gemmatimonadota, Myxococcota, and Firmicutes and increased the relative abundance of Proteobacteria, Acidobacteriota, and Chloroflexi. The results indicate that all four afforestation measures increased the relative abundance of Acidobacteriota and reduced the relative abundances of Gemmatimonadota and Myxococcota in the surface soil. Higher relative abundances of Chloroflexi and lower relative abundances of Gemmatimonadota were observed in the 0–10 cm layer compared to the 10–20 cm layer under all four measures.

The heatmap and sample clustering tree analysis (Figure 8B,C) obtained for the soil bacterial community composition at the phylum level indicate that the bacterial phyla compositions in the differently afforested areas and bare soil were similar at both soil depths and suggest only slightly different bacterial community compositions due to the afforestation measures, while a large difference was observed in the LD soil bacterial community composition.

#### 3.5.4. Soil Bacterial Community PCoA Analysis

**Figure 9:**
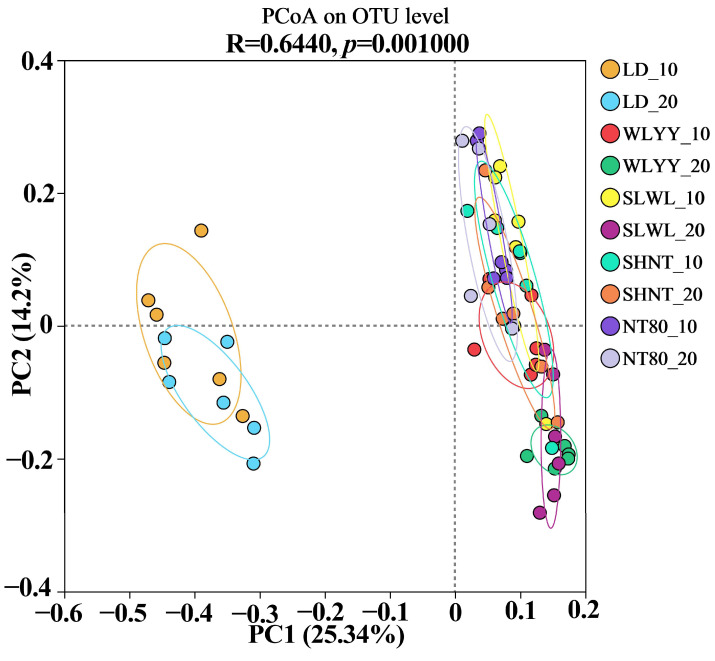
PCoA plot of soil bacterial community composition in differently afforested areas and bare land. LD: bare land containing mobile sand dunes; WLYY: *Salix cheilophila* + *Populus simonii*; SLWL: *Salix psammophila* + *Salix cheilophila*; SHNT: *Artemisia ordosica* + *Caragana korshinskii*; NT80: *Caragana korshinskii*; _10: 0–10 cm soil layer of the sample; _20: 10–20 cm soil layer of the sample.

PCoA analysis was used to analyze the soil bacterial community composition in surface soil samples from areas undergoing the different afforestation measures and from bare land (Figure 9). The closer the samples, the more similar the community composition is. PCoA analysis (R = 0.644, *p* = 0.001) indicated that the bacterial communities in the surface soils of the four afforested areas were relatively clustered, indicating highly similar bacterial communities. The distinct difference observed in the LD results indicates that the bacterial community composition in the LD surface soil differed significantly from that of all four afforested areas, which is consistent with the results obtained from the community heatmap analysis in Section 3.5.3. In the same site, the soil bacterial community composition at both soil depths (0–10 cm and 10–20 cm) showed high similarity in the LD, WLYY, SHNT, and NT80 samples, while the bacterial community composition of the 0–10 cm soil layer in the SLWL samples was less similar to the 10–20 cm layer, indicating that the SLWL measure had a differential effect on the bacterial community composition at different soil depths.

#### 3.5.5. Soil Bacterial Phylum Group Differences

**Figure 10:**
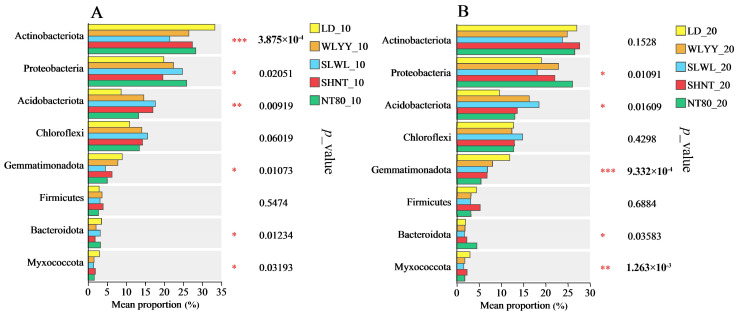
Inter-group difference test of soil bacterial community. Difference test for main bacterial phyla in (**A**) 0–10 cm soil layer and (**B**) 10–20 cm soil layer at different sampling sites. Asterisk indicates that the relative abundance of the same bacterial phyla is significantly different at different sample points: * 0.01 < *p* ≤ 0.05, ** 0.001 < *p* ≤ 0.01, *** *p* ≤ 0.001. LD: bare land containing mobile sand dunes; WLYY: *Salix cheilophila* + *Populus simonii*; SLWL: *Salix psammophila* + *Salix cheilophila*; SHNT: *Artemisia ordosica* + *Caragana korshinskii*; NT80: *Caragana korshinskii*; _10: 0–10 cm soil layer of the sample; _20: 10–20 cm soil layer of the sample.

An inter-group difference test of the major bacterial phyla in the surface soils of the differently afforested areas and bare land showed significant differences in the relative abundance of most major bacterial phyla across the samples (Figure 10). No significant differences were observed in the relative abundance of Chloroflexi and Firmicutes between the samples; however, significant differences were observed in the relative abundances of Proteobacteria, Acidobacteriota, Gemmatimonadota, Bacteroidota, and Myxococcota. Significant differences were observed in the relative abundance of Actinobacteriota in the 0–10 cm soil layer of the different samples.

### 3.6. Correlation Between Soil Physicochemical Properties, Enzyme Activity, and Bacterial Community Structure

#### 3.6.1. Correlation Between Soil Physicochemical Properties and Bacterial Community Structure

**Figure 11:**
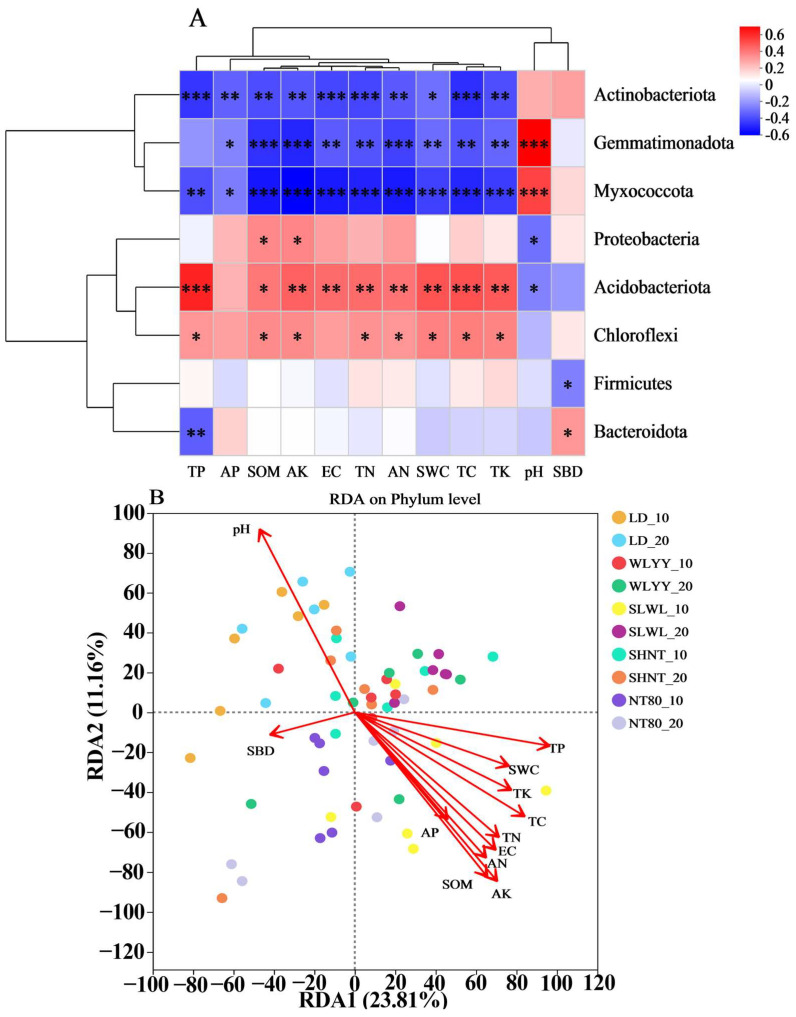
Correlation between soil physicochemical properties and bacterial community structure. (**A**) Correlation heatmap of soil physicochemical properties and relative abundance of major bacterial phyla and (**B**) RDA analysis of soil physicochemical properties and bacterial community structure. Different colors represent positive and negative correlations, with depth of color indicating correlation strength. Asterisks indicate significance: * 0.01 < *p* ≤ 0.05, ** 0.001 < *p* ≤ 0.01, *** *p* ≤ 0.001. EC: electrical conductivity; SBD: soil bulk density; SWC: soil water content; TC: total carbon; TN: total nitrogen; SOM: soil organic matter; TP: total phosphorus; TK: total potassium; AN: alkali-hydrolyzable nitrogen; AP: available phosphorus; AK: available potassium; LD: bare land containing mobile sand dunes; WLYY: *Salix cheilophila* + *Populus simonii*; SLWL: *Salix psammophila* + *Salix cheilophila*; SHNT: *Artemisia ordosica* + *Caragana korshinskii*; NT80: *Caragana korshinskii*; _10: 0–10 cm soil layer of the sample; _20: 10–20 cm soil layer of the sample.

The correlation analysis between the soil physicochemical properties and bacterial community structure in the surface soils from areas under different afforestation measures and from bare land (Figure 11) showed no significant correlation between the relative abundance of Actinobacteriota and pH or SBD (Figure 11A); however, significant negative correlations were observed with other soil physicochemical properties. The relative abundances of Gemmatimonadota and Myxococcota were significantly negatively correlated with AP, SOM, AK, EC, TN, AN, SWC, TC, and TK, and significantly positively correlated with pH. The relative abundance of Myxococcota was also significantly negatively correlated with TP. The relative abundance of Proteobacteria was significantly positively correlated with SOM and AK, and significantly negatively correlated with pH. The relative abundance of Acidobacteriota and Chloroflexi was significantly positively correlated with TP, AP, SOM, AK, TN, AN, SWC, TC, and TK, while Acidobacteriota also showed a significant positive correlation with EC and significant negative correlation with pH. The relative abundance of Firmicutes was significantly negatively correlated only with SBD. The relative abundance of Bacteroidota was significantly negatively correlated with TP and significantly positively correlated with SBD. The analysis of the relationship between soil physicochemical properties and bacterial community structure through RDA can be seen in Figure 11B, with the first and second axes explaining 23.81% and 11.16% of the variation, respectively. The results indicate that, of the soil physicochemical properties, TP was the main factor affecting the bacterial community structure in the surface soils of different afforestation measures and bare land, followed by AK, pH, and TC.

#### 3.6.2. Correlation Between Soil Enzyme Activity and Bacterial Community Structure

**Figure 12:**
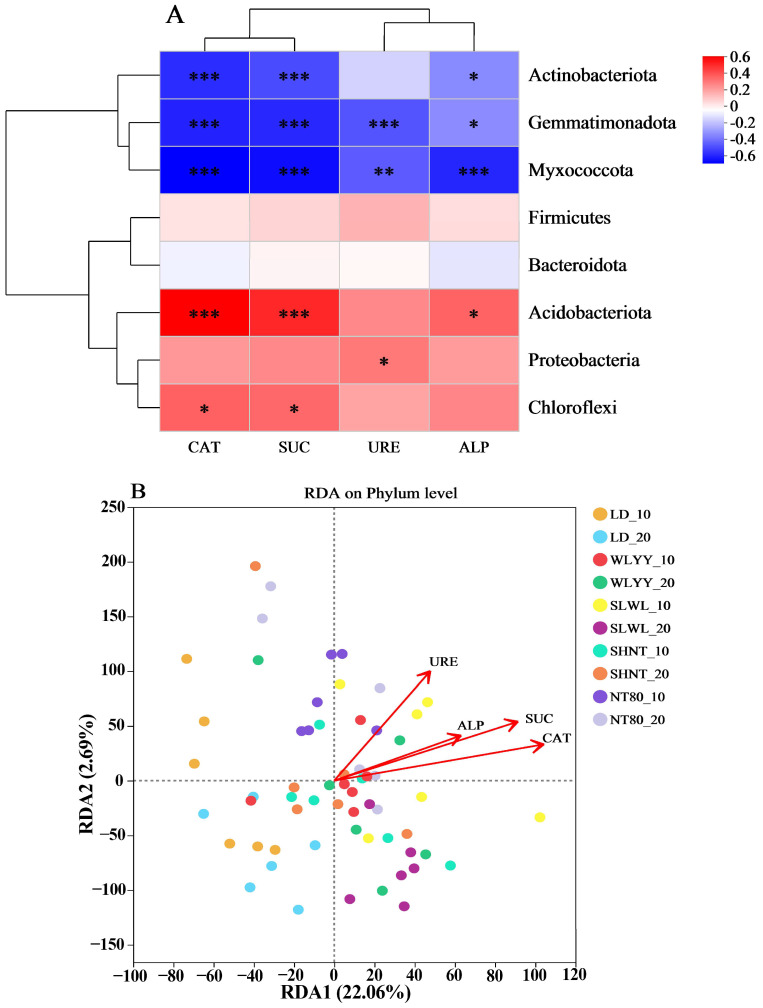
Correlation between soil enzyme activity and bacterial community structure. (**A**) Correlation heatmap between soil enzyme activity and relative abundance of major bacterial phyla, and (**B**) RDA analysis of soil enzyme activity and bacterial community structure. Different colors represent positive and negative correlations, with depth of color indicating correlation strength. Asterisks indicate significance: * 0.01 < *p* ≤ 0.05, ** 0.001 < *p* ≤ 0.01, *** *p* ≤ 0.001. CAT: catalase; SUC: sucrase; URE: urease; ALP: alkaline phosphatase; LD: bare land containing mobile sand dunes; WLYY: *Salix cheilophila* + *Populus simonii*; SLWL: *Salix psammophila* + *Salix cheilophila*; SHNT: *Artemisia ordosica* + *Caragana korshinskii*; NT80: *Caragana korshinskii*; _10: 0–10 cm soil layer of the sample; _20: 10–20 cm soil layer of the sample.

A correlation analysis was also conducted for soil enzyme activity and the bacterial community structure in the surface soils of samples from the areas under different afforestation measures and LD (Figure 12). As shown in Figure 12A, the four soil enzyme activities had a significant impact on the relative abundance of most major bacterial phyla. The relative abundance of Actinobacteriota, Gemmatimonadota, and Myxococcota showed significant negative correlations with CAT, SUC, and ALP, while the relative abundance of Gemmatimonadota and Myxococcota also showed significant negative correlations with URE. The relative abundance of Acidobacteriota was significantly positively correlated with CAT, SUC, and ALP, while Chloroflexi was significantly positively correlated with CAT and SUC and Proteobacteria was significantly positively correlated with URE. No significant correlations were observed between the relative abundance of Firmicutes and Bacteroidota and the four enzymes. An analysis of the relationship between soil physicochemical properties and bacterial community structure using RDA can be seen in Figure 12B, with the first and second axes explaining 22.06 and 2.69% of the variation, respectively. The results indicated that CAT is the key enzyme influencing the bacterial community structure in the surface soils of the differently afforested areas and of bare land, followed by SUC.

## 4. Discussion

### 4.1. Effects of Different Afforestation Measures on the Surface Soil Particle Composition

The composition of soil particles can reflect soil texture and indicate the degree of soil degradation and erosion susceptibility. It has important ecological significance because it affects water retention characteristics, heat preservation, temperature conduction, and the nutrient contents of the soil [25,26]. The alpine sandy land in the Gonghe Basin is severely affected by wind erosion, and without any desertification control measures, wind and sand activities intensify soil erosion, reducing the number of fine particles in the surface soil. In the LD sites of this study, the surface soil particle composition was mainly sand, mainly because a lack of sand barriers and plant coverage leads to poor topsoil stability, resulting in wind and water erosion processes carrying away significant amounts of clay and silt from the surface [27]. The sand content of the surface soil in the areas subjected to afforestation measures was significantly decreased, with increased clay and silt contents. These results align with those of Li et al. [28], who studied the effect of afforestation measures of different ages on the soil particle composition of surface soils in the Tengger Desert, and found that the vegetation in afforested areas can disperse the wind energy near the soil surface, reducing the speed of sand flows, and weakening the sand-carrying effect of the wind. Additionally, the presence of litter on the soil surface can further reduce the direct wind erosion of the soil [29,30,31]. An increase in clay and silt content can improve the degree of soil polymerization and structural stability and enable the soil to more easily retain water and nutrients, which is conducive to the growth and development of plants. These changes have a profound impact on the stability and fertility of the alpine sandy soil [32,33]. Of the afforestation measures investigated in this study, SLWL and WLYY treatment resulted in significantly lower sand content than SHNT and NT80, indicating that SLWL and WLYY are more effective in improving the soil particle composition. Compared to the 10–20 cm soil layer, the 0–10 cm soil layer in the surface soil of the afforested areas showed decreased sand content and increased clay and silt content. Li et al. [34] also found that the soil particle refinement effect decreases with soil depth under different afforestation measures, which is because finer particles in the 0–10 cm soil layer are influenced by the leaching effects of precipitation, and coarse particles in the 10–20 cm layer are affected by biochemical processes via plant root exudates, which thereby changes particle composition in the deeper soil layers. However, this is a long-term process [35]. The results indicate that the four afforestation measures investigated in this study significantly improved the surface soil particle composition of alpine sandy land, particularly the 0–10 cm layer, with the SLWL and WLYY measures being the most effective.

### 4.2. Impact of Different Afforestation Measures on Soil Physicochemical Properties and Enzyme Activity

Soil pH can directly affect the nutrient cycle, fertility, and carbon fixation processes of soil [36]. The surface soil in the study area is alkaline, and compared to the LD, the four afforestation measures reduced the surface soil pH. This suggests that afforestation measures can slow soil alkalization in alpine sandy land, increase soil water retention capacity, and improve wind and sand fixation capacity, thus preventing land degradation [37]. SBD can be used to evaluate soil quality, as it affects soil erosion resistance and significantly impacts soil nutrients [38]. The SHNT measure significantly reduced the surface SBD, while WLYY and SLWL significantly reduced the bulk density in the 10–20 cm soil layer. NT80 had a smaller impact on SBD, which may be due to the different development of plant roots in the surface soil under the various afforestation measures [39] leading to variation in the SBD in the differently afforested areas. SLWL significantly increased the surface SWC, while WLYY, SHNT, and NT80 significantly increased the SWC in the 0–10 cm soil layer. These results align with those obtained by Su et al. [8], while Wang et al. [40] and Xi et al. [41] found that the surface SWC was lower in *Haloxylon ammodendron* plantation forests than in the LD. This difference may be due to the various afforestation measures or the longer recovery periods seen in this study, and the results of this study suggest that the four afforestation measures improved the water retention capacity of the surface soil, especially SLWL, for which the best surface soil water retention capacity was observed. All four afforestation measures increased the EC of the surface soil, similar to the findings of Chang et al. [42]. This is because the surface soil in afforested areas is covered with more litter, and as the litter decomposes, the organic matter in the surface soil increases and ions from the organic matter enter the soil solution, increasing the EC [43]. Figure 4 confirms the significant positive correlation between soil organic matter and EC.

Soil nutrient content is an important indicator of soil fertility and is mainly derived from the decomposition of plant litter; thus, this factor is greatly influenced by vegetation type [44]. Increasing soil nutrient content can improve soil structure, promote the recovery and development of microbial activities and plant communities, and enhance the ecosystem’s stability and ability to resist external disturbances [45]. For example, increasing the content of soil nitrogen, phosphorus, and potassium can not only improve the photosynthetic performance of plants and promote plant growth but also promote the accumulation of soluble protein and soluble sugar in plant cells, which is of great significance for maintaining the osmotic pressure of cells [46]. All four afforestation measures significantly increased the surface soil nutrient content, similar to the findings of Li et al. [47]. Among the four measures, SLWL showed the greatest increase in the soil nutrient content, followed by WLYY. Only SLWL was associated with an increase in the AP content, which may be because the root exudates of *S. psammophila* or *S. cheilophila* significantly enhance the AP content of the soil [48], or may be because the surface soil moisture in the SLWL measure area may be more suitable for microbial activity, promoting the release of organic phosphorus [49]. Soil enzymes can influence soil fertility, promote soil biochemical reactions, and drive nutrient cycling and energy transformation in the soil [50]. All four afforestation measures increased the CAT, SUC, URE, and ALP activity in the surface soil, similar to the findings of Huang et al. [51]. This increase in enzyme activity is because the increased nutrient content in the surface soil promotes the activity of relevant enzymes [52]. The extent of the enzyme activity increase varied with the different afforestation measures, with the SLWL measure showing a greater increase in CAT and SUC activity and the SHNT and WLYY measures showing a greater increase in URE and ALP activity. Qian et al. [53] found that, after 17 years of vegetation restoration in Mu Us sandy land, the enzyme activity of the 0–10 cm soil layer was higher than that of deeper soil layers. This is similar to the results of the present study, with the soil nutrient content in the 0–10 cm soil layer exceeding that in the 10–20 cm layer, demonstrating a surface aggregation effect [54]. The results indicate that the four afforestation measures in this study significantly impacted physicochemical properties and enzyme activity in the surface soil, improving the soil quality, with SLWL having the greatest improvement effect, followed by WLYY.

However, it should be noted that the implementation of afforestation measures will increase the EC of sandy soil, which is not conducive to the colonization and breeding of non-halophytic herbs. In addition, our study was limited by the lack of physicochemical properties data of surface soil since 1980, meaning that the long-term effects of afforestation measures on soil could not be fully explored.

### 4.3. Effects of Different Afforestation Measures on the Surface Soil Bacterial Community Structure

Soil microorganisms play a significant role in soil ecosystems [55], improving soil permeability and increasing its nutrient content and fertility, thereby influencing the soil structure. The sensitivity of soil microorganisms to environmental changes means that they can act as indicators of soil quality changes [56,57]. Among the soil microorganisms, bacteria account for the largest proportion, with diverse species and strong adaptability rendering them dominant in soil microbial communities; thus, they are widely used as important indicators for evaluating soil quality [58,59]. In the study area, the dominant bacterial phyla in the surface soil were Actinobacteriota, Proteobacteria, Acidobacteriota, and Chloroflexi, which is similar to the dominant phyla found in other sandy surface soils [60,61]. Diversity index analysis revealed that afforestation measures can enhance the bacterial community richness in the surface soil, leading to more effective organic matter decomposition and improved soil fertility and nutrient utilization. In addition, bacterial richness was higher in the 0–10 cm soil layer than in the 10–20 cm layer, consistent with the findings of Wang et al. [24] and Wang et al. [62], driven by the higher nutrient content in the surface soil of the afforested areas. Additionally, the nutrient content in the soil layers exhibits a surface aggregation effect, leading to lower bacterial richness in the deeper layers [63]. The bacterial community diversity was higher in the 0–10 cm soil layer than in the LD in all four afforested areas, with the community heatmap and PCoA analysis also indicating significant differences in the bacterial community composition of the afforested areas and the LD.

Moreover, the relative abundances of most major bacterial phyla varied significantly across the sites, suggesting that the four afforestation measures had a significant impact on the bacterial community structure of the surface soil. This variation is primarily attributed to differences in the physical and chemical properties of soil, which affect the growth and reproduction of soil bacterial communities. Actinobacteriota and Proteobacteria were the most abundant bacterial phyla in the study area and play a key role in the soil nutrient cycle [64]. Actinobacteriota, known for their strong ability to decompose complex organic matter [65], accelerate the decomposition of organic matter and release of nutrients in the soil in the area with afforestation measures, where litter is more distributed. This helps improve soil quality and may promote plant growth and ecosystem restoration. Proteobacteria, which can adapt to different environmental conditions through various forms and metabolic pathways [66], help alpine sandy soils maintain certain life activities and metabolic functions. All four afforestation measures significantly increased the relative abundance of Acidobacteria, which is also mainly involved in the degradation of plant residue polymers, and there was also a higher amount of litter in the afforested areas [67]. This will lead to rapid accumulation of organic matter in the soil in the area of afforestation measures, thus further improving soil quality. Chloroflexi is involved in driving the cycling of C, N, S, and other ecosystem materials [68], while Gemmatimonadota can convert sugar molecules into vitamins [69]. Compared to the 10–20 cm layer, the relative abundance of Chloroflexi was higher and that of Gemmatimonadota was lower in the 0–10 cm soil layer, indicating more active ecosystem material cycling in this layer, possibly leading to higher vitamin content.

The bacterial community structure is highly sensitive to changes in the soil environment. Tian et al. [70] found that pH is a key factor affecting bacterial community structure in Mu Us sandy land, while Cao et al. [60] suggested that SOM is the most important environmental factor influencing the bacterial community structure in the soil of the Pinus sylvestris forest in Horqin sandy land. In this study, TP was found to be the key factor influencing the bacterial community structure in the surface soil of the differently afforested areas and LD, significantly affecting the relative abundance of most major bacterial phyla. The main factors leading to the inconsistency between this study and previous studies may be the higher altitude, lower temperatures, or the longer afforestation recovery period in the study area. Soil bacteria are key participants in the decomposition process, influencing enzyme activity [71]. In this study, the activity of all four studied soil enzymes was significantly correlated with the relative abundance of most major bacterial phyla, similar to the findings of previous research [72], further demonstrating the close relationship between soil enzyme activity and the bacterial community structure.

## 5. Conclusions

Compared with the soil in the LD, all four afforestation measures significantly improved the surface soil particle composition, increasing the clay and silt contents. Compared with the LD, all four measures significantly increased the contents of TC, TN, SOM, AN, and AK in surface soil; WLYY, SLWL, and SHNT significantly increased TP and TK; and SLWL significantly increased AK. Among the four measures, compared with the LD, SLWL most significantly increased the activities of catalase and sucrase, SHNT most significantly increased the activity of urease, and WLYY most significantly increased the activity of alkaline phosphatase. Overall, SLWL had the greatest impact on the soil nutrient content and enzyme activity, followed by WLYY. Soil nutrient content and enzyme activity exhibited a surface aggregation effect in the topsoil across all four afforested areas. The four measures significantly affected the surface soil bacterial community structure and may have enhanced the bacterial community richness and diversity in the 0–10 cm soil layer. The bacterial community composition differed markedly from that of the LD, with significant differences in the relative abundance of most major bacterial phyla observed at the various sampling points. The bacterial community richness in the 0–10 cm soil layer was higher than that in the 10–20 cm layer. TP was found to be the key factor influencing the bacterial community structure in the surface soils of areas undergoing different afforestation measures and the LD. The activity of the four soil enzymes was also found to be significantly correlated with the relative abundance of most major bacterial phyla. The results of this study are expected to be of use in the development of improved afforestation measures that can limit desertification in the future. They can provide reference for the prevention and control of desertification in other alpine sandy land.

## 6. Patents

Shaobo Du, Huichun Xie, Chongyi E., Tianyue Zhao, Shuang Ji, Zhiqiang Dong, Shaoxiong Zhang, Haokun Wu. A plant fixation device for desertification control in deserts [P].utility model, 26 July 2024.

## Figures and Tables

**Table 1:** Basic information regarding the sample site.

Plot	Longitude	Latitude	Altitude (m)	Area (m^2^)	Vegetation Coverage	Plant Spacing	Soil Depth (cm)	Number
LD	100°14′28.266″ E	36°13′50.785″ N	2822	273,333	-	-	0~10	LD_10
							10~20	LD_20
WLYY	100°15′19.0″ E	36°15′20.1″ N	2829	2500	82%	1.5 m × 1.5 m	0~10	NL_10
							10~20	NL_20
SLWL	100°14′16.9″ E	36°14′5.43″ N	2824	33,330	69%	1.5 m × 2 m	0~10	SB_10
							10~20	SB_20
SHNT	100°15′8.89″ E	36°14′41.2″ N	2817	19,998	66%	1.5 m × 1.5 m	0~10	NT_10
							10~20	NT_20
NT80	100°14′7.28″ E	36°14′55.7″ N	2,11	10,000	89%	1.5 m × 1.5 m	0~10	SH_10
							10~20	SH_20

Note: ‘-’ indicates meaningless.

## Data Availability

The original contributions presented in the study are included in the article.
